# On the Correlation of Physical Forces: Being the Substance of a Course of Lectures Delivered in the London Institution, in the Year 1843

**Published:** 1847-01

**Authors:** 


ART. X.—*On the Correlation of Physical Forces : being the substance of a Course of Lectures delivered in the London Institution in the year* 1843. By W. R. GROVE, Esq., M.A. F.R.S., Barrister at Law. Published at the request of the Proprietors of the London Institution.—*London,* 1846. Royal 8vo, pp. 52.

THIS pamphlet gives formal expression to a view which has long been gaining ground amongst philosophers in this and other countries, that we must discard altogether the old notion of "imponderable forms of matter," as a clumsy hypothesis invented to elude a difficulty which could not be solved in an earlier condition of scientific inquiry; and that we are to consider light, heat, electricity, magnetism, and chemical affinity, as *affections* of ordinary forms of matter, or manifestations of their properties. And further, that these agencies are all *correlative ;* that is, they possess such a reciprocal dependence, that either of them may, as a force, produce or be convertible into another. We cannot here stop to investigate the principal arguments upon which Mr. Grove bases this conclusion; but we may state that they appear to us to be entirely satisfactory, and that we cannot refer to any brief exposition of the question which gives so lucid and complete a view of it as that which is contained in the pamphlet before us. Since its publication, (as many of our readers are doubtless aware,) a discovery has been announced by Mr. Grove himself, which goes very much to strengthen his argument; namely, that heat, like electricity, has the power of *decomposing* water, as well as of causing the union of its elements, according to the mode in which it is applied. For if a pure platinum wire, immersed in water, be kept at the highest temperature at which it can be maintained, short of fusion, the surrounding liquid will be resolved into its constituent gases, just as when a galvanic current is passed through it. And yet at a temperature a very little lower, the very same heated wire would cause the reunion of the two gases which it has separated, in the same manner as they may be made to combine by transmitting an electric spark through the mixture.

On one point Mr. Grove has gone beyond other philosophers who have taken up the same line of speculative reasoning; for he has included motion among the agencies which are thus correlated. At first sight it might appear to be altogether removed from them; but he gives some very ingenious arguments, founded upon the phenomena of friction, to the effect that motion, if checked, produces heat; whilst, as is well known, heat produces motion. Thus, if these two agencies be mutually convertible, the correlation of motion with all the rest is established, through the intermediation of heat. Further, when friction takes place between heterogeneous substances, electricity is developed; and thus another link is afforded between motion and the other agencies, any of which may be excited by electricity, and most of them by heat.

"I have said that either of the forces above mentioned may, *mediately* or *immediately,* produce the others; and this is all I can venture to predicate of them in the present state of science; but I will venture as an opinion, founded after much consideration, that science is rapidly progressing towards the establishment of immediate or direct relations between all these forces. Where at present no immediate relation is established between any of them, electricity generally forms the intervening link or middle term." (p. 13.)

In regard to light, Mr. Grove embraces a view which was long since suggested by the eminent Euler, and which appears well worthy of being thoroughly tested by mathematical research; namely, that light is not, as in the Newtonian hypothesis, an emanation of material but imponderable particles; nor, as supposed by the partisans of the undulatory theory, the result of the vibrations of a hypothetical ether; but that it results from a vibration of the molecules of matter itself, just as sound is propagated by the vibrations of wood, or as waves are by water. The striking analogies between sound and light, as regards their mode of transmission, are all in favour of such a view: and it is no objection to it to say that a vacuum still permits the transmission of light; since it may safely be affirmed that no real vacuum has ever been produced; and there is fully as much reason to presume the existence, even in the spaces that intervene amongst the heavenly bodies, of matter (in the ordinary sense of the term) in a very finely-divided condition, as there is to suppose that this space is occupied by the luminous particles of Newton's imponderable, or by the luminiferous ether of more recent times.

Altogether this outline of Mr. Grove's views is one which is most fertile in topics of interesting speculation; whilst it shows how closely such speculations may be connected, by a logical mind, with phenomena obvious to our senses. We commend its study, to those who are capable of mastering it, as an example of the mode in which we trust that the phenomena of vitality may be ultimately generalized; when the same preparation shall have been made, by patient and accurate research, for the development of analogous views.

